# Intraoperative ultrasound reduces the need for re-excision in breast-conserving surgery

**DOI:** 10.1186/s12957-015-0731-2

**Published:** 2015-11-24

**Authors:** Hasan Karanlik, Ilker Ozgur, Dilek Sahin, Merdan Fayda, Semen Onder, Ekrem Yavuz

**Affiliations:** Surgical Oncology Unit, Institute of Oncology, Istanbul University, Istanbul, Turkey; Department of General Surgery, Acibadem International Hospital, Bakirkoy, Istanbul Turkey; Department of Radiology, Institute of Oncology, Istanbul University, Istanbul, Turkey; Department of Radiation Oncology, Istanbul Medical Faculty, Istanbul University, Istanbul, Turkey; Department of Pathology, Istanbul Medical Faculty, Istanbul University, Istanbul, Turkey

**Keywords:** Ultrasound-guided lumpectomy, Intraoperative ultrasound, Positive margin, Re-excision rate

## Abstract

**Background:**

The purpose of this study was to evaluate ultrasound-guided surgery for palpable breast cancer by comparing the standard palpation-guided surgery in terms of the extent of healthy breast tissue resection, the percentage of tumor-free margins, and cosmetic outcomes.

**Methods:**

This was a prospective, observational cohort study conducted from January 2009 to July 2011. Breast cancer patients, diagnosed via biopsy, were operated in guidance with either ultrasound or palpation. Patient demographics, tumor features, intraoperative findings, pathologic and cosmetic results, intraoperative-measured ultrasound margins, and pathology margins were compared.

**Results:**

Ultrasound (US)-guided lumpectomy was performed on 84 women and palpation-guided lumpectomy on 80 women. Patient demographics and tumor characteristics showed no differences. The rate of re-excision was 17 % for the palpation-guided surgery group, and 6 % for the US-guided group (*p* = 0.03). There was good correlation between the closest margins recorded by US and pathology margins (*r* = 0.76, *p* = 0.01). Volume of resection was significantly larger in the palpation-guided group despite the similar size of tumors (*p* = 0.048). Cosmetic outcome of surgery was equivalent between groups.

**Conclusions:**

Intraoperative ultrasound guidance for excision of palpable breast cancers is feasible and gives results in terms of pathologic margins that are comparable with those achieved by standard palpation-guided excisions.

## Background

With improvement in detection of breast cancer at an earlier stage, more breast cancers are being detected at a smaller size. This makes breast conservation surgery (BCS) a feasible and preferred option for many women diagnosed with early-stage breast cancer. Many women choose to pursue BCS because of the improved cosmetic and psychological outcome from breast preservation. Local recurrence after BCS is related to patient age, tumor size and grade, and presence of multifocal or multicentric disease [1–3]. However, margin status is the strongest predictor of local recurrence [3–6]. Although local recurrence risk is reduced by wider tissue excision, cosmetic results are adversely affected by more extensive operations. Evaluation of the resection margins is commonly undertaken as an aid to achieve an optimal balance between adequate local control and cosmetic results. Some authors reported cosmetic results after BCS as unacceptable in up to 30 % of patients. The main factor determining the cosmetic result is resection volume. If it is larger than 50–85 cm^3^_,_ the cosmetic result is purposeful to be a failure [7–12].

Several techniques have been suggested in order to avoid inadequate margins in breast-conserving surgery [4, 13]. These include gross examination of the lumpectomy specimen, frozen section, touch prep analysis, intraoperative specimen radiography, and intraoperative ultrasound (US) as well as newer investigational tools [13–15]. There have been several groups who have published their experiences with lumpectomy using the intraoperative US technique [7, 13, 16–22]. Intraoperative US for palpable breast cancers not only facilitates clearer margins with fewer additional treatment interventions but also effects decision-making by achieving optimum resection volume which end up with good cosmetic results. US guidance offers some benefits over needle localization guidance in terms of non-palpable breast cancer. US guidance provides better operational anatomy coordination which causes a surgeon to end up with better margin clearance and less re-excision rates and also better cosmetic outcomes. Palpable breast cancer patients should benefit from US-guided lumpectomy as it was reported for non-palpable breast cancer patients as the surgeons reduces the rate of positive margins. Some studies were reported to have better surgical accuracy in the guidance of US than palpation for palpable breast cancers [7, 13, 23].

We hypothesized that intraoperative US would confer upon the surgeon a greater ability to discern the precise margins of the tumor. This would enable a more fitting initial excisional diameter and fewer close positive margins, thereby reducing the need for subsequent interventions. Furthermore, we hypothesized that other benefits accrued by use of intraoperative US include minimization of normal breast parenchyma resection and improved cosmetic results due to optimal initial breast incision placement.

## Methods

### Patients

This was an institutional review board-approved, prospective, observational cohort study conducted from January 2009 to July 2011. Beginning in June 2010, US technology which was not available before became available in our operating room. We prospectively evaluated 84 consecutive patients undergoing US-guided lumpectomy for a palpable breast cancer after June 2010. We selected 80 consecutive palpable breast cancer patients operated at our institution without any guidance technique before introduction of intraoperative US from our data.

All patients in both groups were diagnosed via core biopsy as having invasive breast cancer. All admitted breast cancer patients’ data, findings and results are recorded at our institution. Patients who may have undergone subsequent mastectomy as a second operation were not excluded from the initial analysis. Although patient allocation into either cohort was non-randomized, we did include all eligible patients and do not believe that there are any factors of significance that would bias this sample. Patients who received neoadjuvant chemotherapy, diagnosed following previous excisional biopsy, and patients with non-palpable tumors were excluded from the study.

### Surgery

Surgery was performed under general anesthesia by an experienced oncological breast surgeon. The surgery started with the axillary procedure. The sentinel node was sent for frozen section study, and if metastasis was diagnosed, axillary lymph node dissection was performed during the same procedure. Node-positive patients who were pre-operatively confirmed by US-guided fine-needle aspiration biopsy underwent axillary lymph node dissection. After the axillary procedure, surgery of the breast was performed.

### Palpation-guided surgery

In the palpation-guided surgery group, tumor excision was guided by the palpation of the surgeon in the standard manner. The index finger was used to palpate the mass, retract it, and guide the dissection. In this procedure, the adequacy of the resection was based on the experience of the surgeon without objective imaging during surgery. The aim of surgical excision was to obtain a 1-cm rim of healthy adjacent breast tissue around the malignant breast lesion. Tumor was excised in a cylindrical manner with muscular fascia as posterior margin. At the time of operation, specimens were sent to the pathology department for margin analysis but not to the radiology department for specimen radiology. Intraoperative re-excision was performed if the specimen margin was reported to be positive or less than 5 mm macroscopically. This is a routine margin approach procedure applied at our institution [24].

### Ultrasound-guided surgery

Intraoperative localization was performed using a multifrequency 10-MHz linear array ultrasound probe. At the beginning of the study, for the first ten procedures, an experienced breast radiologist was present and assisted the procedure in the operating room. Later on, our experience developed, and a radiologist’s assistance was not required.

**Fig. 1:**
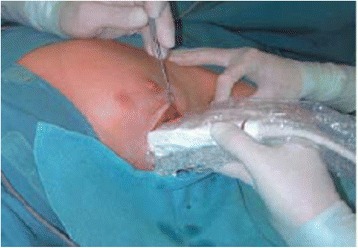
Intraoperative tumor localization by ultrasound

Tumor localization of was done after probe’s midpoint is placed transversely just over the tumor center. The probe’s ends were marked over the skin. These two spots were connected with a line. Later, the procedure was repeated in sagittal direction. Little pressure was used to avoid tissue displacement. Planed surgical incision and extent of the planed excision tissue with a 1-cm clear border away from the tumor was marked as well. The provided information by the US for the depth of the tumor from skin and distance to muscular layer was used to estimate flaps thickness (Fig. 1).

**Fig. 2:**
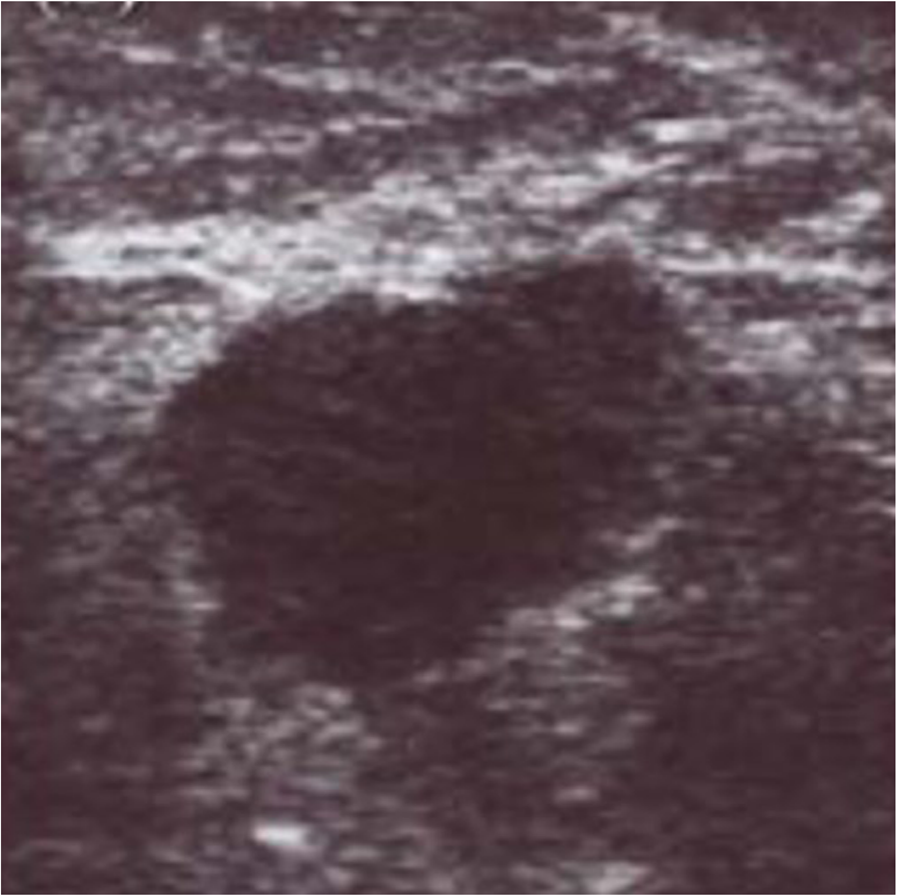
Ultrasound image of the tumor

**Fig. 3:**
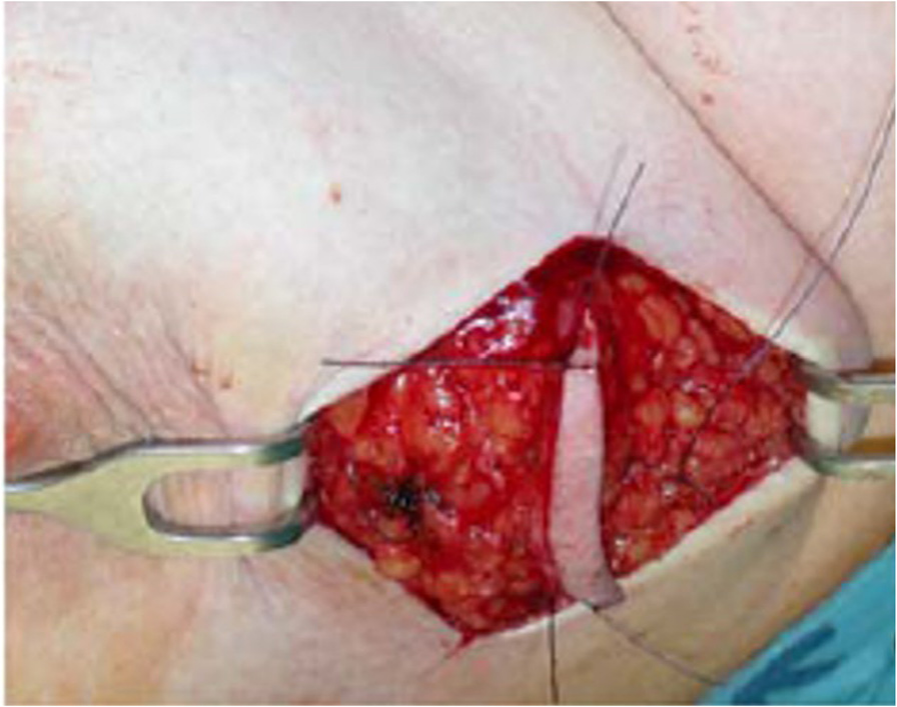
Marking of the tumor

**Fig. 4:**
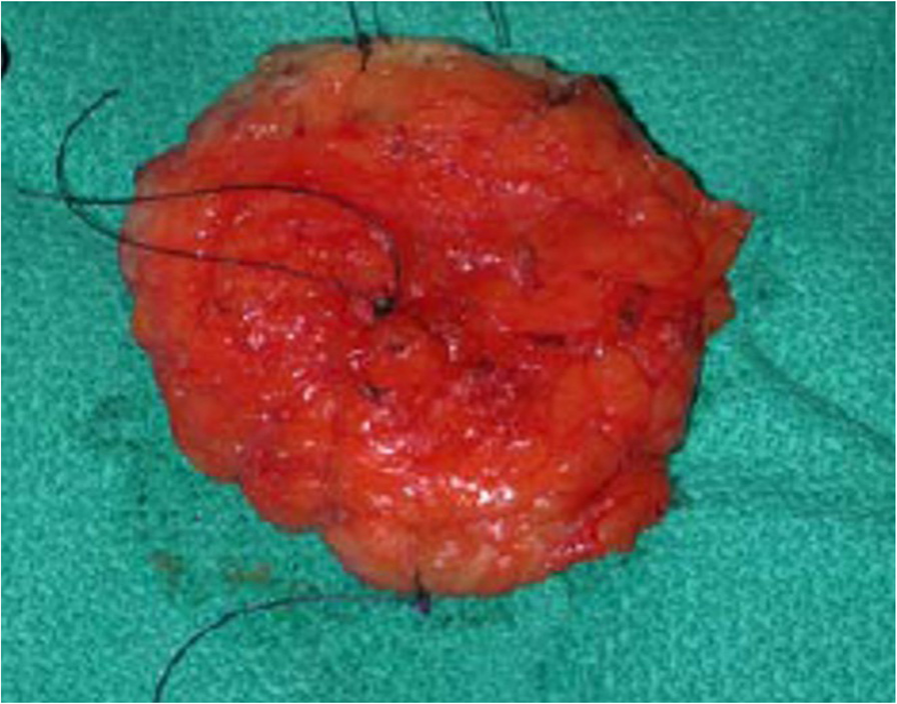
Excised and oriented tumor

**Fig. 5:**
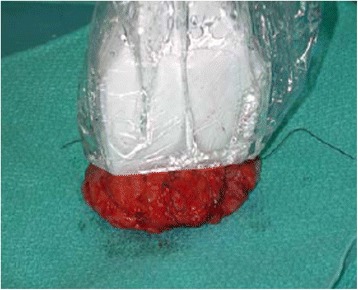
Ex vivo ultrasound of the tumor

After marked skin incision, skin flaps were prepared till the previously marked tissue excision border (Fig. 2). The tumor was confirmed via US to still be in the excision border with clear margins (Fig. 3). Then, tissue was excised in a cylindrical manner perpendicularly to the chest wall (Fig. 4). Posterior adequate margin was deep enough to the muscular fascia even containing the fascia itself. Then, specimen margins were marked, and ex vivo US were performed to determine tumor-free margins (Fig. 5). If a margin less than or equal to 5 mm was detected, a re-excision of that area was performed.

Direct volume measurements were performed by using a water displacement technique undertaken by the same team member (breast care nurse) in the operating room on each occasion which is performed as a standard procedure due to ongoing studies since 2008. The excised breast tissue was placed in a cylinder of known volume. The specimen was then submerged in the cylinder, and the volume of the displaced water was measured. The validity and reproducibility of this method was confirmed by submerging breast implants of known volume into the cylinder and assessing the water volume displaced.

After specimen fixation in formalin solution, all specimens were evaluated for tumor size in length, width, and height using a millimeter ruler and microscopic margin status by histopathologic evaluation of permanent sections. Tumor volume was calculated using length × width × height formula. The closest margin of excision was measured and documented. Any patients with microscopically positive margins (tumor cells present at an inked margin) were re-excised in a second operative procedure.

### Data analysis

Demographic, clinical, and pathological data including age, body mass index (BMI), menopausal status, tumor localization, histopathologic type, T and N stage, grade (Scarf-Bloom**-**Richardson), the presence of intraductal component, lymphovascular invasion and necrosis, and receptor status (ER, PR, HER2-neu) were gathered. Intraoperative data including length of surgery, ultrasound findings, and the rate of intraoperative re-excision were recorded. For cosmetic evaluation, patients and/or patients’ pictures were re-evaluated at 6 months postoperatively and classified by two clinicians. These clinicians were not informed of the patients’ therapeutic procedure, and they used the four category scale: excellent, good, fair and poor according to the Harvard Cosmetic Scale [25].

Microsoft Excel software (Microsoft Luxembourg S.a.r.l., 20 Rue Eugene Ruppert, Luxembourg) was used to record the data. Comparisons between continuous variables were analyzed using the *t* test or Mann-Whitney *U* test. Comparisons between categorical variables were based on Pearson’s chi-square test of Fisher’s exact test. Pearson’s correlation coefficient was used to assess the correlation between the US findings and margin width. In all statistical analyses, a *p* value of <0.05 was considered significant. Data analysis was carried out with the Statistical Package for Social Sciences (SPSS) version 15.0 (SPSS, Chicago, IL, USA).

## Results

The study was designed in two groups for palpable breast cancer, US-guided lumpectomy group and palpation-guided lumpectomy group which consisted of consecutive 84 and 80 patients, respectively. Patient and tumor characteristics are listed in Table 1. None of the screening items exhibited differences between the two groups.

**Table 1:** Comparison of patient and tumor characteristics between palpation-guided and US-guided lumpectomy groups

Characteristic	Palpation-guided lumpectomy	US-guided lumpectomy	*p* value
Age, mean (SD), y	51.1 (12.8)	53.4 (13.3)	0.25
Body mass index, mean (SD)	25.2 (4.5)	25.1 (3.5)	0.8
Menopausal status			
Premenopausal	37 (46 %)	30 (36 %)	
Postmenopausal	43 (54 %)	54 (64 %)	0.2
Tumor location			
Outer	59 (74 %)	56 (67 %)	
Inner	21 (26 %)	28 (33 %)	0.39
Tumor size			
1–10 mm	6 (7 %)	11 (13 %)	
11–20 mm	31 (39 %)	29 (35 %)	
>20 mm	43 (54 %)	44 (52 %)	0.26
Lymph node status			
Negative	49 (61 %)	54 (64 %)	
Positive	31 (39 %)	30 (36 %)	0.74
Grade			
1	12 (15 %)	10 (12 %)	
2	36 (45 %)	44 (52 %)	
3	32 (40 %)	30 (36 %)	0.62
EIC^a^			
Yes	26 (33 %)	22 (26 %)	
No	54 (67 %)	62 (74 %)	0.39
Lymphovascular invasion			
Yes	45 (56 %)	50 (60 %)	
No	35 (44 %)	34 (40 %)	0.75
Multifocality			
Yes	17 (21 %)	11 (13 %)	
No	63 (79 %)	73 (87 %)	0.21
ER			
Positive	64 (80 %)	73 (87 %)	
Negative	16 (20 %)	11 (13 %)	0.29
PR			
Positive	54 (67 %)	65 (77 %)	
Negative	26 (33 %)	19 (23 %)	0.17
HER2-neu status			
Amplified	15 (19 %)	15 (18 %)	
Nonamplified	65 (81 %)	69 (82 %)	0.88

*EIC* extensive intraductal component

The re-excision rates were not statistically significant in the US-guided group between the first, second, third, and fourth quartile of patients. Positive margins were observed in five (6 %) of the patients in the US-guided group and 14 (17 %) of the patients in the palpation-guided group (*p* = 0.03). Three (60 %) patients’ re-excision specimens were reported to have residual ductal carcinoma in situ or invasive ductal carcinoma and two (40 %) patients’ re-excision specimens had only post-operative changes without any residual atypia or malignancy in the US-guided group. Seven (50 %) patients had residual ductal carcinoma in situ or invasive ductal carcinoma out of 14 patients undergoing re-excision in the palpation-guided group. Residual disease rate in the re-excision pathology was not statistically different between two groups (*p* = 0.9), (Table 2).

**Table 2:** Comparisons of main pathological outcome measures and findings between palpation-guided and US-guided lumpectomy groups

Characteristic	Palpation-guided lumpectomy	US-guided lumpectomy	*p* value
Tumor size, mean (SD)	24.9 (12.2)	22.8 (10.8)	0.24
Resection volume at first excision, cubic centimeter, (SD)	108.1 (63.4)	89.9 (53.9)	0.048
Intraoperative re-excision			
Yes	30 (37 %)	24 (29 %)	
No	50 (63 %)	60 (71 %)	0.25
Need for second operation to achieve adequate margins			
Yes	14 (17 %)	5 (6 %)	
No	66 (83 %)	79 (94 %)	0.03
Tumor identified at intraoperative re-excision specimen			
Yes	5 (17 %)	7 (29 %)	
No	25 (83 %)	17 (71 %)	0.33
Tumor identified at second operation specimen			
Yes	7 (50 %)	3 (60 %)	
No	7 (50 %)	2 (40 %)	0.9

Shaving rates (intraoperative re-excision) of the US-guided lumpectomy group was 29 % due to close margins on US of the lumpectomy specimen. The palpation-guided surgery group exhibited a higher frequency (37 %) of shaving rates (*p* = 0.25). Among patients that underwent additional shave margins, seven within the US-guided group (29 %) were spared additional surgery thanks to more precise discernment capabilities provided by the US apparatus. Under normal conditions, due to the close or positive margins on the lumpectomy specimen, these seven patients would have been required to undergo additional surgery. This included two patients with margins of 3–4 mm, three patients with margins of ≤2 mm, and two patients with involved margins according to the specimen ultrasonography. The significant correlation between US findings and margin positivity can be seen in Table 3 (*r* = 0.76, *p* = 0.01).

**Table 3:** Correlation between US findings and pathological margin involvement in US-guided lumpectomy group (*r* = 0.76, *p* = 0.01)

US findings	Number of patients	Involved margin (pathologically)
Adequate margins (≥5 mm)	60	4 (7 %)
Close margins (3–4 mm)	12	2 (17 %)
Minimally involved margins (≤2 mm)	8	3 (38 %)
Involved margins	4	2 (50 %)

The tumor size was 2.5 ± 1.2 cm for palpation-guided lumpectomy versus 2.3 ± 1.1 cm for the US-guided lumpectomy group (*p* = 0.24). Comparing resection volume with calculations from the detailed post-operative pathology reports and volume measurements, we found that the volume of resection was significantly larger in the palpation-guided group despite the similar size of tumor (*p* = 0.048). The tumor/specimen volume in the palpation-guided and US-guided lumpectomy groups was 0.09 and 0.097, respectively. Also, tumor/specimen + re-excision volume was not different between two groups (*p* > 0.05). Cosmetic outcome of surgery was equivalent between groups (*p* = 0.79).Of the 60 patients, 55 (92 %) in the palpation-guided lumpectomy group and 67 of 71 patients (94 %) in the US-guided group have a post-operative cosmetic rating of good or excellent.

## Discussion

Because of the numerous disadvantages of needle localization, several authors investigated the use of intraoperative US in an effort to avoid the use of needle localization of non-palpable breast lesions [26–29]. Some authors reported that US-guided excision of non-palpable breast masses is practicable and even superior to needle localization excision [7, 13, 16, 17, 20, 22]. Rahusen et al. [19] prospectively evaluated 19 patients with 20 mammographically nonpapable lesions. They used a 10-MHz transducer during surgery to localize the tumor and plan the excision. They then compared their experience with 43 wire localization excisions performed during the same period. In the wire excision group, only 17 of 43 (40 %) resections were deemed to have “adequate margins”. In the excisions using ultrasound guidance, 17 (89 %) had acceptable margins. Similarly, in their small randomized study, they recruited 49 patients, and negative margins were reported in 89 % of the US-guided excisions compared with 55 % of the needle localization-guided excisions [16]. They found an advantage in US-guided excision for obtaining adequate margins in their studies. They were assisted with an experienced radiologist in the operating room. In addition to positive margin rates, some drawbacks have been reported with needle localization, including miss rates of 0–22 %, wire transection, dislocation, or migration, and scheduling difficulties. Also, the patient may experience the anxiety and discomfort of having a needle/wire in the breast while awaiting the surgical procedure. Vasovagal reactions also have been reported in approximately 20 % of cases [30, 31]. Krekel and colleagues [32] also demonstrated a reduced level of involved margins in their multicenter, randomized, and controlled study designed for US-guided surgery versus palpation-guided surgery in palpable breast cancer patients. Of the 65 patients, 2 (3 %) patients allocating ultrasound-guided surgery had tumor-involved margins compared with 12 (17 %) of the 69 patients who were assigned palpation-guided surgery (difference 14 %, 95 % CI 4–25; *p* = 0.0093). Seven (11 %) patients who received US-guided surgery and 19 (28 %) of those who received palpation-guided surgery required additional treatment (*p* = 0.015). Compared with palpation-guided surgery, ultrasound-guided surgery can significantly lower the proportion of tumor-involved resection margins, thus reducing the need for re-excision, mastectomy, and radiotherapy boost. They stated that optimum resection volumes influence unnecessary resection and could contribute to improved cosmetic results and quality of life.

The technique of US guidance for palpable masses is mentioned in the literature. Many surgeons may feel halfhearted to use US for a procedure instead of their palpation senses. This could be due to unwillingness in practical terms or due to unavailability. Krekel [33] stated that skilled surgeons can gain the expertise needed to do US-guided surgery in a fairly short training period of up to eight procedures. Intraoperative US was not available in our operating theater either until 2010. Palpable lesions are reported to be associated with higher margin positivity rates than non-palpable lesions, so US should be more promising with palpable lesions. Despite this, there are few publications on US-guided breast-conserving surgery in the literature. In the randomized study of Moore et al. [23], they compared 27 patients undergoing US guidance and 24 patients undergoing palpation guidance for palpable breast cancers. They found that the rate of positive margins was significantly less for the US-guided group (3 %) compared with the palpation-guided group (29 %). They stated that US guidance resulted in a decreased re-excision rate and improved cosmesis. In our study, adequate resection was performed on 94 % of patients in the US-guided surgery group and on 83 % of patients in the palpation-guided group (*p* = 0.03). Ultrasound correctly identified the closest pathology margin in 85 % of patients. We accepted tumor cells at an inked margin to perform re-excision at a second operative procedure. However, the intention was usually to achieve a 1-cm margin by ultrasound, and intraoperative re-excision of the surgical cavity was carried out for ultrasound margins of <5 mm in every case.

Another technique for evaluation of surgical margins is intraoperative specimen radiography. If microcalcifications occur close to the edges of the specimen, the associated cavity edges may be shaved to remove any residual malignant disease. Specimen radiography is found to be reliable in identifying clear margins (74 % positive predictive value) and reduces the rate of reintervention from 31 to 20 % [34]. However, the use of radiographic X-ray mammography is limited due to limitations in detecting small, noncalcified lesions and a high rate of nonspecific findings [35]. Lee and Carter [36] examined postexcision specimen radiographs of 125 patients and found sensitivity for detecting margin positivity as 49 %. They concluded that intraoperative specimen radiography could not be relied on solely but presents a valuable addition to BCS. We did not perform specimen radiology because we investigated the value of intraoperative US impact on reducing the marginal positivity status alone. Further studies may be designed to compare both techniques.

On the other hand, ultrasound overestimated the pathology margins in most cases. Margin overestimation by ultrasound may lead the surgeon to incorrectly believe that the excised margins are inappropriate. The overestimation of the majority of the tumor margins may be explained in part by the tendency of ultrasound to underestimate the pathologic tumor diameter [28]. Another possible factor that needs to be taken into account as a cause for overestimation of tumor margins by ultrasound is the compression of the specimen by the ultrasound probe during ex vivo examination of the margins. The axial margins are then further away from the less compressible tumor. This suggests that we cannot rely solely on ultrasound to determine the extent of resection and that palpation may still play an important part in margin assessment. However, a measured ultrasound margin of ≥0.5 cm resulted in 93 % adequate margins (Table 3). Increasing the threshold for re-resection could theoretically improve the rate of adequate excisions, but this would lead to a significant increase in re-excisions in patients in whom the original lumpectomy will prove to be pathologically adequate.

US guidance gives the advantage of resecting less normal tissue while maintaining the clear margins with both palpable and non-palpable masses [7, 13, 22, 23]. Moore et al. [23] found that the volume of the lumpectomy specimen for palpable infiltrating ductal carcinomas was smaller in their US-guided group (104 cm^3^) versus their palpation-guided group (114 cm^3^), with increased mean clear margin width (7.6 versus 4.8 mm, respectively). Also Krekel et al. [32] reported that US-guided surgery resulted in smaller excision volumes compared to palpation-guided surgery in palpable breast cancer patients (38 versus 57 cm^3^; *p* = 0.002). Larger excisions may lead to less margin positivity but may end up with poor cosmesis. Our data show that US-guided lumpectomy achieved good margins with a much smaller volume of resection compared with palpation-guided surgery (mean resection volume, 89.9 versus 108.1 cm^3^, respectively, *p* = 0.048). Since the tumor/specimen volume was nearly the same in both groups; attention should be given on the point that intraoperative US can significantly lower the rate of positive margins without unnecessary tissue resection, not only on the resected tissue amount. In our study, additional shave margins were taken in nearly one-third of the patients undergoing US-guided lumpectomy. Twenty-nine percent of these patients who underwent intraoperative re-excision according to the ultrasonographic findings have been found to have a residual tumor in their cavity-shaving specimen. Sonographic assessment in the operating room provides appropriate assessment of all margins to plan excision, and specimen ultrasonography can guide immediately if a margin revision is indicated.

The same surgeon performed all lumpectomies. Although we did not find any statistically significant differences in the re-excision rates between the first 20 and last 20 cases, we experienced a learning curve at the beginning due to a new procedure in use which was not demonstrated by the re-excision data. Intraoperative and specimen US provide useful information to the surgeon for incision site and extension of margin shaving.

## Conclusions

In conclusion, comparing complete tumor excision results between US-guided and palpation-guided lumpectomy for palpable breast cancer US provides fair enough or even better results. US also provides real-time localization of palpable tumors leading to optimal incision and extent of excision. Intraoperative US may cause reduction in terms of positive margins which may lead to a reduction in need of margin-shaving rates and tumor bed radiotherapy boost. Just as US has become a standard tool of the breast surgeon’s office practice, we propose a role for US in the accurate intraoperative assessment of palpable breast tumors as well.

## Ethical approval and consent to participate

This study meets the standards of ethical approval of the University of Istanbul. This study was presented at the eighth European Breast Cancer Conference (EBCC-8) held in Vienna in 2012.
